# Effect of Sex and Flow Status on Outcomes After Surgical or Transcatheter Aortic Valve Replacement

**DOI:** 10.1016/j.jacadv.2024.100853

**Published:** 2024-02-16

**Authors:** Rasmus Carter-Storch, Rebecca T. Hahn, Amr E. Abbas, Melissa A. Daubert, Pamela S. Douglas, Sammy Elmariah, Yanglu Zhao, Michael J. Mack, Martin B. Leon, Philippe Pibarot, Marie-Annick Clavel

**Affiliations:** a Institut Universitaire de Cardiologie et de Pneumologie de Québec/Quebec Heart and Lung Institute, Laval University, Quebec, Quebec, Canada; b Department of Cardiology, Odense University Hospital, Odense, Denmark; c Department of Medicine, Columbia University Irving Medical Center, New York, New York, USA; d Corewell Health, William Beaumont University Hospital, Royal Oak, Michigan, USA; e Duke University, Durham, North Carolina, USA; f Massachusetts General Hospital, Boston, Massachusetts, USA; g Edwards Lifesciences, Irvine, California, USA; h Baylor Scott & White Health, Plano, Texas, USA; i Columbia University, New York, New York, USA

**Keywords:** low flow aortic stenosis, SAVR, sex-differences, TAVR

## Abstract

**Background:**

Low stroke volume index <35 ml/m^2^ despite preserved ejection fraction (paradoxical low flow [PLF]) is associated with adverse outcomes in patients with aortic stenosis undergoing transcatheter aortic valve replacement (TAVR) or surgical aortic valve replacement (SAVR). However, whether the risk associated with PLF is similar in both sexes is unknown.

**Objectives:**

The purpose of this study was to analyze the risk associated with PLF in severe aortic stenosis for men and women randomized to TAVR or SAVR.

**Methods:**

Patients with ejection fraction ≥50% from the PARTNER (Placement of Aortic Transcatheter Valves) 2 and 3 trials were stratified by sex and treatment arm. The impact of PLF on the 2-year occurrence of the composite of death or heart failure hospitalization (primary endpoint) and of all-cause mortality alone (secondary endpoint) was analyzed. Analysis of variance was used to assess baseline differences between groups. Multivariate Cox regression analysis was used to identify predictors of the endpoint.

**Results:**

Out of 2,242 patients, PLF was present in 390 men and 239 women (30% vs 26%, *P* = 0.06). PLF was associated with a higher rate of NYHA functional class III to IV dyspnea (60% vs 54%, *P* < 0.001) and a higher prevalence of atrial fibrillation (39% vs 24%, *P* < 0.001). PLF was a significant predictor of the primary endpoint among women undergoing SAVR in multivariate analysis (adjusted HR: 2.25 [95% CI: 1.14-4.43], *P* = 0.02) but was not associated with a worse outcome in any of the other groups (all *P* > 0.05).

**Conclusions:**

In women with PLF, TAVR may improve outcomes compared to SAVR. PLF appears to have less impact on outcomes in men.

Since the inception of transcatheter aortic valve replacement (TAVR) 30 years ago,^1^ it has proven to be a noninferior or superior alternative to surgical aortic valve replacement (SAVR) among patients with severe aortic stenosis (AS). Although short- and medium-term outcomes are comparable between the 2 treatment modalities for patients at low and intermediate risk,^2^^,^^3^ long-term outcomes for TAVR are still uncertain, and TAVR is therefore primarily used in patients with shorter life expectancy or intermediate/high surgical risk, after individual heart team evaluation.^4^^,^^5^

Although AS was previously thought to be similar in men and women, several sex-specific studies have shown that there are differences between the sexes in pathophysiology, epidemiology, presentation of AS, and outcome after aortic valve replacement (AVR).^6^ Among high-risk patients in the PARTNER (Placement of Aortic Transcatheter Valves) 1 trial, women had significantly reduced 2-year survival with SAVR compared to TAVR.^7^ Whether this applies to low- and intermediate-risk patients is unknown, but in an analysis of the PARTNER 2, SAPIEN 3 cohort of high- and intermediate-risk patients, neither the combined nor individual risk cohorts showed sex differences in mortality or disabling stroke out to 1 year.^8^ Moreover, patients with AS often present with a low flow state, defined as a stroke volume index (SVi) <35 ml/m^2^, either with reduced (classical low flow) or preserved (paradoxical low flow [PLF]) left ventricular (LV) ejection fraction (LVEF). PLF has been shown to be associated with increased mortality compared to patients with normal flow (NF) after AVR.^9^, ^10^, ^11^ PLF is usually more prevalent among women,^10^^,^^12^ but whether the prognostic implication of PLF is the same in men vs women after AVR is unknown.

The aim of this study was, therefore, to investigate the prognostic implication of PLF among men and women after TAVR and SAVR among patients with low and intermediate surgical risk enrolled in the PARTNER 2 and 3 trials.

## Methods

### Study sample

The PARTNER 2 and 3 trials were multicenter, randomized clinical trials comparing TAVR and SAVR among patients with intermediate operative risk (PARTNER 2: Society of Thoracic Surgeons score 4%-8%)^2^ and low operative risk (PARTNER 3: Society of Thoracic Surgeons score <4%).^3^ All patients had severe AS, with aortic valve area (AVA) ≤0.8 or 1.0 cm^2^ in the 2 trials, respectively, and mean gradient ≥40 mm Hg. Patients in PARTNER 2 were randomly assigned to SAVR with any commercially available bioprosthetic surgical valve or TAVR with an Edwards SAPIEN XT, in PARTNER 3 to SAVR with any commercially available bioprosthetic surgical valve or TAVR with an Edwards SAPIEN 3 transcatheter heart valve. Full lists of inclusion and exclusion criteria have been previously published.^2^^,^^3^ The PARTNER trials were approved by the Institutional Review Board at each site, and written informed consent was obtained from all the patients.

In the present analysis, we excluded patients with reduced LVEF <50%, as well as patients with missing data for AVA, SVi, mean gradient, or LVEF.

### Echocardiography

Echocardiograms were analyzed at a core laboratory blinded to randomization and clinical endpoints, as previously described.^13^ LV dimensions and mass, as well as biplane Simpson LVEF, were estimated as recommended by the American Society of Echocardiography guidelines for chamber quantification.^14^ Peak aortic jet velocity was measured at the window of maximal velocity, and mean gradient was estimated using the simplified Bernoulli equation. Stroke volume was calculated by multiplying the LV outflow tract diameter and Doppler velocity time integral measured just proximal to the native annulus. AVA was calculated from the continuity equation and indexed to body surface area (AVAi).^15^ Valvular regurgitations at baseline were assessed and graded as recommended by the American Society of Echocardiography.^16^ Patients were dichotomized according to guidelines as NF: SVi ≥35 ml/m^2^ or PLF: SVi <35 ml/m^2^.^5^

Patient-prosthesis mismatch (PPM) was defined using the normal reference value of AVA (for the model and size of the implanted prosthesis) indexed to the patient’s body surface area, with thresholds adjusted for obese patients as per guidelines.^17^

Postoperative echocardiograms performed 1 month after AVR were analyzed for the presence of transvalvular or paravalvular regurgitation and graded according to American Society of Echocardiography guidelines.^16^ Mean gradient, maximal jet velocity, and AVA were also measured at 1 month.

### Procedure

Details on the procedure for implantation of the Edwards SAPIEN XT^18^ and the SAPIEN 3^19^ TAVR systems have been previously published. Procedures were performed via transfemoral or alternative access, depending on preprocedural assessment. Dual antiplatelet therapy with clopidogrel and aspirin was recommended for at least 1 month after TAVR.

### Study endpoints

The primary endpoint in this substudy was the composite of death from any cause or rehospitalization for heart failure symptoms within 2 years. The secondary endpoint was all-cause mortality within 2 years. Time to event was defined as the time to the first endpoint within 2 years; for censored cases, time to event was either the time to the last participation date, or 730 days, whichever was shorter. A clinical events committee independently adjudicated all potential events. Clinical outcomes were reported as defined by the Valve Academic Research Consortium-2 definitions.^20^

### Statistics

Analysis of variance (ANOVA) for continuous variables and chi-square tests for categorical variables were used to compare patient demographics, procedure information, and echo parameters among ≥3 groups; Student’s *t*-test and chi-square test were used to compare continuous variables and categorical variables, respectively, between 2 groups. Kaplan-Meier curves and log-rank tests were used to compare the primary and secondary endpoints by treatment and SVi group within each sex. Multivariable Cox regression models were used to further identify predictors of endpoints from among the following covariates: SVi, age, sex, coronary artery disease, NYHA functional class, hypertension, diabetes, atrial fibrillation, chronic obstructive pulmonary disease, chronic kidney disease, treatment arm (SAVR/TAVR), LVEF, mean gradient, patient-prosthesis mismatch, and transvalvular regurgitation at 30 days.

All statistical analyses were done using SAS version 9.4 (SAS institute). A 2-sided *P* value <0.05 was considered statistically significant.

## Results

Out of a total of 2,953 patients (PARTNER-2 SAVR: n = 936, PARTNER-2 TAVR: n = 1,069, PARTNER-3: 948), 711 patients were excluded due to LVEF <50% or missing echo data. Of the 2,242 patients remaining in this study, 1,321 (59%) were men and 921 (41%) were women. There were 629 patients (28%) with PLF, with a numerically higher proportion of PLF among men than women (30% vs 26%, *P* = 0.06).

### Baseline characteristics according to sex and flow

Women in this study, compared with men, were older, and had higher NYHA functional class and lower Kansas City Cardiomyopathy Questionnaire score at baseline. They had lower prevalence of coronary artery disease, diabetes, and atrial fibrillation, but more often had chronic kidney disease and frailty indicators. On echocardiography, women’s SVi and AVAi were similar to that of men. Women had higher LVEF, but a higher prevalence of ≥ moderate mitral and tricuspid regurgitation (Table 1, Supplemental Table 1).

**Table 1:** Clinical and Echocardiographic Characteristics According to Sex and Flow Profile

		Men (N = 1,321)			Women (N = 921)			
	Total (N = 2,242)	SVi <35 (n = 390)	SVi ≥35 (n = 931)	Within Men *P* Value	SVi <35 (n = 239)	SVi ≥35 (n = 682)	Within Women *P* Value	ANOVA *P* Value
Clinical parameters								
Age (y)	78.9 ± 7.5	78.4 ± 7.9	78.3 ± 7.5	0.8572	79.6 ± 7.2	79.9 ± 7.1	0.5706	<0.0001
Body mass index (kg/m^2^)	29.4 ± 5.9	30.5 ± 5.5	28.8 ± 4.8	<0.0001	31.1 ± 7.4	29.0 ± 6.7	<0.0001	<0.0001
Body surface area (m^2^)	1.9 ± 0.2	2.1 ± 0.2	2.0 ± 0.2	<0.0001	1.8 ± 0.2	1.8 ± 0.2	<0.0001	<0.0001
STS score (%)	4.2 (2.2, 5.6)	4.2 (2.2, 5.6)	3.4 (1.7, 5.1)	<0.0001	4.7 (3.4, 6.3)	4.7 (2.7, 6.1)	0.4457	<0.0001
NYHA functional class I	8 (0.4%)	1 (0.3%)	5 (0.5%)	0.0437	0 (0.0%)	2 (0.3%)	0.0195	<0.0001
NYHA functional class II	1,008 (45.0%)	169 (43.3%)	471 (50.6%)		79 (33.1%)	289 (42.4%)		
NYHA functional class III	1,024 (45.7%)	177 (45.4%)	382 (41.1%)		129 (54.0%)	336 (49.3%)		
NYHA functional class IV	201 (9.0%)	43 (11.0%)	72 (7.7%)		31 (13.0%)	55 (8.1%)		
KCCQ overall summary score	62.5 (44.8, 80.0)	61.1 (43.2, 76.8)	68.0 (51.0, 84.7)	<0.0001	53.4 (37.8, 70.4)	58.7 (42.7, 77.0)	0.0104	<0.0001
Diabetes mellitus	715 (31.9%)	162 (41.5%)	290 (31.1%)	0.0003	82 (34.3%)	181 (26.6%)	0.0229	<0.0001
Coronary artery disease	1,147 (51.2%)	239 (61.3%)	529 (56.9%)	0.1448	107 (44.8%)	272 (39.9%)	0.1864	<0.0001
Atrial fibrillation	626 (27.9%)	169 (43.3%)	246 (26.5%)	<0.0001	74 (31.0%)	137 (20.1%)	0.0006	<0.0001
Chronic obstructive pulmonary disease	452 (20.2%)	99 (25.4%)	180 (19.4%)	0.0155	44 (18.4%)	129 (19.0%)	0.8341	0.048
Creatinine >2 mg/dL	83 (3.7%)	11 (2.8%)	42 (4.5%)	0.1532	7 (2.9%)	23 (3.4%)	0.7396	0.37
Frailty	80 (3.6%)	8 (2.1%)	22 (2.4%)	0.7334	13 (5.4%)	37 (5.4%)	0.9971	0.001
AV hemodynamics								
AV mean gradient (mm Hg)	47.4 ± 12.3	44.2 ± 10.6	48.3 ± 12.0	<0.0001	45.8 ± 13.1	48.7 ± 12.8	0.0026	<0.0001
AV peak jet (cm/s)	442.0 ± 52.8	426.3 ± 46.8	445.9 ± 50.9	<0.0001	434.4 ± 56.9	448.3 ± 55.1	0.0009	<0.0001
AV area index (cm^2^/m^2^)	0.4 ± 0.1	0.3 ± 0.1	0.4 ± 0.1	<0.0001	0.3 ± 0.1	0.4 ± 0.1	<0.0001	<0.0001
LV systolic structure and function								
SVi (mL/m^2^)	40.3 ± 8.7	30.7 ± 3.4	43.9 ± 7.2	<0.0001	30.7 ± 3.23	44.3 ± 7.0	<0.0001	<0.0001
Heart rate	68 ± 12	72 ± 13	64 ± 10	<0.0001	77 ± 12	69 ± 11	<0.0001	<0.0001
LV ejection fraction (%)	64.0 ± 7.3	61.6 ± 6.9	64.1 ± 6.9	<0.0001	62.9 ± 7.8	65.6 ± 7.4	<0.0001	<0.0001
LV end-diastolic diameter (mm)	4.7 ± 0.6	4.8 ± 0.5	5.0 ± 0.5	<0.0001	4.3 ± 0.5	4.5 ± 0.5	0.0003	<0.0001
LV end-systolic diameter (mm)	3.0 ± 0.6	3.1 ± 0.6	3.1 ± 0.6	0.1883	2.8 ± 0.6	2.7 ± 0.5	0.5329	<0.0001
LV end-diastolic volume (mL)	99.8 ± 28.9	103.6 ± 25.7	113.4 ± 28.9	<0.0001	76.9 ± 20.1	85.9 ± 21.2	<0.0001	<0.0001
LV end-systolic volume (mL)	35.7 ± 14.2	39.5 ± 13.4	40.5 ± 14.9	0.2628	28.2 ± 10.9	29.0 ± 10.6	0.3202	<0.0001
LV mass index (g/m^2^)	198.8 ± 108.2	203.7 ± 106.2	201.6 ± 118.2	0.7715	193.2 ± 98.3	194.1 ± 97.5	0.9080	0.3804
Relative wall thickness	0.5 (0.4-0.5)	0.5 (0.4-0.5)	0.4 (0.4-0.5)	<0.0001	0.5 (0.4-0.6)	0.5 (0.4-0.5)	0.0002	<0.0001
Concomitant valve regurgitations								
Moderate + mitral regurgitation	154 (7.0%)	25 (6.7%)	46 (5.0%)	0.2420	25 (10.8%)	58 (8.7%)	0.3424	0.004
Moderate + aortic regurgitation	149 (6.7%)	11 (2.9%)	73 (7.9%)	0.0007	7 (3.0%)	58 (8.6%)	0.0042	0.0002
Moderate + tricuspid regurgitation	155 (7.3%)	27 (7.5%)	47 (5.3%)	0.1202	30 (13.3%)	51 (7.8%)	0.0138	0.0004

Values are mean ± SD, median (Q1, Q3), or n (%). ANOVA was used for continuous variables. Chi-square tests were used for categorical variables. An expanded table is presented as in Supplemental Table 3.

AV = aortic valve; KCCQ = Kansas City Cardiomyopathy Questionnaire; LV = left ventricular; NYHA = New York Heart Association Class dyspnea symptoms; STS = Society of Thoracic Surgeons; SVi = stroke volume index.

Compared to patients with NF, patients with PLF had a higher STS score, higher NYHA functional class, lower Kansas City Cardiomyopathy Questionnaire score, higher prevalence of coronary artery disease and diabetes mellitus, higher heart rate, and higher prevalence of pacemaker and atrial fibrillation (Supplemental Table 2). These findings were similar when flow was further stratified by sex (Supplemental Table 3). On preprocedural echocardiographic examination, patients with PLF also had lower AVAi, SVi, and transvalvular gradient, smaller LV dimensions and lower LVEF. This was especially the case among women with PLF, who had the smallest LV end-diastolic volumes. Patients with PLF had more mitral and tricuspid regurgitation, but had less aortic regurgitation (Table 1, Supplemental Table 2.1).

### Procedural and postprocedural data

Procedural and postprocedural data according to sex and preprocedural SVi are presented in Table 2 for SAVR and Table 3 for TAVR. During both TAVR and SAVR, women received smaller sized valves than men. For SAVR patients, 22% of women and only 1% of the men received a valve size ≤19 mm. This difference was even more pronounced in the subgroup of women with PLF (Table 2). For TAVR patients, similar findings were observed with 9.6% of women and <0.1% of men receiving a 20 mm valve size, and again women with PLF more often received smaller valves compared to women with NF (Table 3).

**Table 2:** Procedural Information by Sex and SVi in SAVR Group

		Men (N = 590)			Women (N = 434)			
	Total (N = 1,024)	SVi <35 (n = 422)	SVi ≥35 (n = 168)	Within Men *P* Value	SVi <35 (n = 310)	SVi ≥35 (n = 124)	Within Women *P* Value	ANOVA *P* Value
Valve size								
17 mm	1 (0.1%)	0 (0.0%)	0 (0.0%)	0.2775	0 (0.0%)	1 (0.3%)	0.1785	<0.0001
19 mm	98 (9.6%)	1 (0.6%)	1 (0.2%)		35 (28.2%)	61 (19.7%)		
21 mm	288 (28.1%)	20 (11.9%)	56 (13.3%)		63 (50.8%)	149 (48.1%)		
23 mm	362 (35.4%)	69 (41.1%)	183 (43.4%)		23 (18.5%)	87 (28.1%)		
25 mm	220 (21.5%)	68 (40.5%)	139 (32.9%)		3 (2.4%)	10 (3.2%)		
27 mm	49 (4.8%)	10 (6.0%)	37 (8.8%)		0 (0.0%)	2 (0.6%)		
29 mm	6 (0.6%)	0 (0.0%)	6 (1.4%)		0 (0.0%)	0 (0.0%)		
Days in hospital	8.0 (6.0, 11.0)	8.0 (6.0, 10.0)	8.0 (6.0, 10.0)	0.3763	8.0 (7.0, 12.0)	9.0 (7.0, 12.0)	0.4594	0.02
Days in ICU	3.0 (2.0, 5.0)	3.0 (2.0, 6.0)	3.0 (2.0, 5.0)	0.1955	3.0 (2.0, 5.0)	4.0 (2.0, 6.0)	0.7886	0.27
Discharged to home?	589 (57.6%)	100 (59.5%)	287 (68.0%)	0.0502	61 (49.6%)	141 (45.5%)	0.4395	<0.0001
30 d echo information								
AV mean gradient (mm Hg)	11.1 ± 4.29	10.2 ± 3.94	10.8 ± 4.06	0.1370	11.6 ± 4.42	11.8 ± 4.58	0.5853	0.0003
AV peak velocity (cm/s)	227.3 ± 42.7	217.2 ± 41.9	224.1 ± 40.4	0.0793	232.0 ± 43.6	235.5 ± 44.5	0.4924	<0.0001
AV area (cm^2^)	1.6 ± 0.44	1.7 ± 0.43	1.8 ± 0.41	0.0018	1.3 ± 0.34	1.5 ± 0.38	<0.0001	<0.0001
AV area index (cm^2^/m^2^)	0.8 ± 0.22	0.8 ± 0.20	0.9 ± 0.21	<0.0001	0.7 ± 0.20	0.8 ± 0.23	<0.0001	<0.0001
PPM	362 (43.0%)	73 (52.5%)	112 (32.6%)	<0.0001	63 (62.4%)	114 (44.2%)	0.0019	<0.0001

Values are n (%), median (Q1, Q3), or mean ± SD. ANOVA was used for continuous variables. Chi-square tests were used for categorical variables.

AV = aortic valve; ICU = intensive care unit; PPM = patient-prosthesis mismatch; SAVR = surgical aortic valve replacement; SVi = stroke volume index.

**Table 3:** Procedural Information by Sex and SVi in TAVR Group

		Men (N = 590)			Women (N = 487)			
	Total (N = 1,218)	SVi <35 (N = 509)	SVi ≥35 (n = 222)	Within Men *P* Value	SVi <35 (n = 372)	SVi ≥35 (n = 115)	Within Women *P* Value	*P* Value
Approach								
Transfemoral	1,133 (93.0%)	204 (91.9%)	474 (93.1%)	0.8071	110 (95.7%)	345 (92.7%)	0.1837	0.69
Transapical	60 (4.9%)	13 (5.9%)	24 (4.7%)		2 (1.7%)	21 (5.6%)		
Other	25 (2.1%)	5 (2.3%)	11 (2.2%)		3 (2.6%)	6 (1.6%)		
Valve size								
20 mm	48 (3.9%)	0 (0.0%)	1 (0.2%)	0.0608	15 (13.0%)	32 (8.6%)	0.4549	<0.0001
23 mm	417 (34.2%)	37 (16.7%)	54 (10.6%)		71 (61.7%)	255 (68.5%)		
26 mm	548 (45.0%)	134 (60.4%)	304 (59.7%)		28 (24.3%)	82 (22.0%)		
29 mm	205 (16.8%)	51 (23.0%)	150 (29.5%)		1 (0.9%)	3 (0.8%)		
Days in hospital	4.0 (3.0, 5.0)	3.0 (3.0, 5.0)	3.0 (3.0, 4.0)	0.7330	4.0 (3.0, 5.0)	4.0 (3.0, 6.0)	0.8521	0.07
Days in ICU	2.0 (2.0, 3.0)	2.0 (2.0, 2.0)	2.0 (1.0, 3.0)	0.7779	2.0 (1.0, 3.0)	2.0 (2.0, 3.0)	0.8738	0.06
Discharged to home?	1,089 (89.4%)	203 (91.4%)	477 (93.7%)	0.2676	97 (84.3%)	312 (83.9%)	0.9030	<0.0001
30 d echo information								
AV mean gradient (mm Hg)	11.7 ± 4.7	10.9 ± 4.0	12.1 ± 4.9	0.0015	11.3 ± 4.2	13.3 ± 5.5	0.0005	<0.0001
AV peak velocity (cm/s)	230.9 ± 43.0	222.4 ± 38.0	233.7 ± 41.6	0.0007	226.0 ± 41.6	242.8 ± 46.2	0.0006	<0.0001
AV area (cm^2^)	1.7 ± 0.4	1.7 ± 0.3	1.9 ± 0.3	<0.0001	1.5 ± 0.3	1.5 ± 0.3	0.1064	<0.0001
AV area index (cm^2^/m^2^)	0.9 ± 0.2	0.8 ± 0.2	0.9 ± 0.2	<0.0001	0.8 ± 0.2	0.9 ± 0.2	0.0008	<0.0001
PPM	724 (36.8%)	84 (41.0%)	124 (26.0%)	<0.0001	51 (47.2%)	103 (30.9%)	0.0020	<0.0001

Values are n (%), median (Q1, Q3), or mean ± SD. ANOVA was used for continuous variables. Chi-square tests were used for categorical variables.

AV = aortic valve; ICU = intensive care unit; PPM = patient-prosthesis mismatch; SVi = stroke volume index; TAVR = transcatheter aortic valve replacement.

Women had longer hospital stays after the procedure and were less frequently discharged to their homes. The 30-day mortality rate for the whole sample was numerically higher without statistical significance among women with NF and PLF (2.2 and 2.5%) compared to men with NF and PLF (1.2 and 1.3%) (ANOVA *P* = 0.26).

At the 1-month echocardiogram, patients with PLF had smaller AVAi despite lower mean gradients, and more often had patient-prosthesis mismatch (Tables 2 and 3). This was most pronounced for women with PLF undergoing SAVR, where 63 (62%) had patient-prosthesis mismatch (Table 2).

### Association of PLF and sex with outcomes

During a median follow-up of 2 (IQR: 2-2) years, there were 180 deaths and 103 hospitalizations for heart failure.

**Figure 1:**
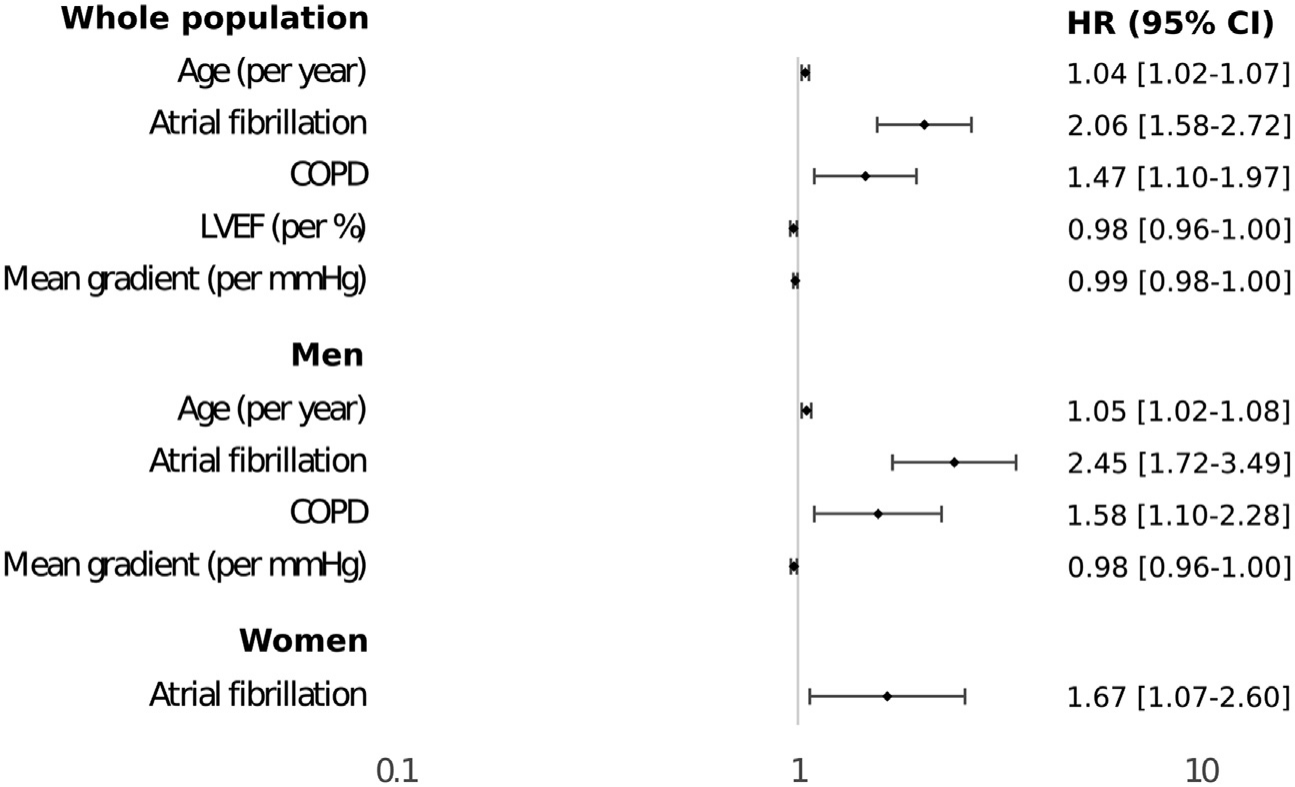
**Forest Plot of Significant Multivariable Predictors of Death or Rehospitalization for Heart Failure in Whole Sample** Multivariable cox regression analysis. Variables are the only significant multivariable predictors of the combined primary endpoint. The following covariates were added in the model: Age, sex, coronary artery disease, NYHA functional class, hypertension, diabetes, atrial fibrillation, COPD, chronic kidney disease, treatment (SAVR/TAVR), LVEF, stroke volume index, mean gradient, patient-prosthesis mismatch, and transvalvular regurgitation at 30 days. The full multivariable analyses results are available as Supplemental Tables 4 to 8. COPD = chronic obstructive pulmonary disease; LVEF = left ventricular ejection fraction; SAVR = surgical aortic valve replacement; TAVR = transcatheter aortic valve replacement.

In the whole sample, significant predictors of the primary endpoint were age, atrial fibrillation, chronic obstructive pulmonary disease, lower LVEF, and lower mean gradient prior to the procedure. In men, the same variables, except LVEF, were predictors of the endpoint, while in women atrial fibrillation was the only significant independent predictor (Figure 1).

Low preprocedural SVi was not a predictor of the primary endpoint in the whole cohort, in women or men separately, or in the subgroup of TAVR patients (Supplemental Tables 5 and 6). However, among patients randomized to SAVR, low SVi was associated with the primary outcome in women (univariable HR: 1.98 [95% CI: 1.09-3.62], *P* = 0.03) but not in men (HR: 1.20 [95% CI: 0.68-2.13], *P* = 0.40) (Supplemental Tables 7 and 8). Among women undergoing SAVR, after adjusting for known risk factors (age, coronary artery disease, NYHA functional class, hypertension, diabetes, atrial fibrillation, chronic obstructive pulmonary disease, chronic kidney disease, LVEF, preprocedural mean gradient, patient-prosthesis mismatch, and transvalvular regurgitation at 30 days), PLF was the only independent predictor of the primary outcome (adjusted HR: 2.25 [95% CI: 1.14-4.43], *P* = 0.02) (Table 4, Central Illustration).

**Table 4:** Multivariable Cox Model for Death/Heart Failure Hospitalization (Female SAVR Patients)

	HR (95% CI)	*P* Value
SVi <35 mL/m^2^	2.25 (1.14-4.43)	0.02
Age, per 1 y increase	1.02 (0.96-1.08)	0.53
Coronary artery disease	1.05 (0.56-1.97)	0.88
NYHA functional class III/IV (dyspnea symptoms)	0.80 (0.39-1.63)	0.54
Hypertension	3.31 (0.45-24.51)	0.24
Diabetes	1.11 (0.56-2.22)	0.77
Atrial fibrillation	1.51 (0.76-2.96)	0.24
Chronic obstructive pulmonary disease	1.73 (0.80-3.75)	0.17
Chronic kidney disease moderately to severely decreased or poorer	0.73 (0.33-1.61)	0.43
LVEF, per 1% increase	0.96 (0.91-1.01)	0.14
Aortic mean gradient, per 1 mm Hg increase	1.01 (0.99-1.03)	0.40
Patient-prothesis mismatch	0.80 (0.40-1.61)	0.54
Transvalvular regurgitation at 30 d ≥ mild	2.86 (0.37-22.17)	0.31

30-day landmark multivariable Cox regression.

HF = heart failure; LVEF = left ventricular ejection fraction; SAVR = surgical aortic valve replacement; SVi = stroke volume index.

**Central Illustration:**
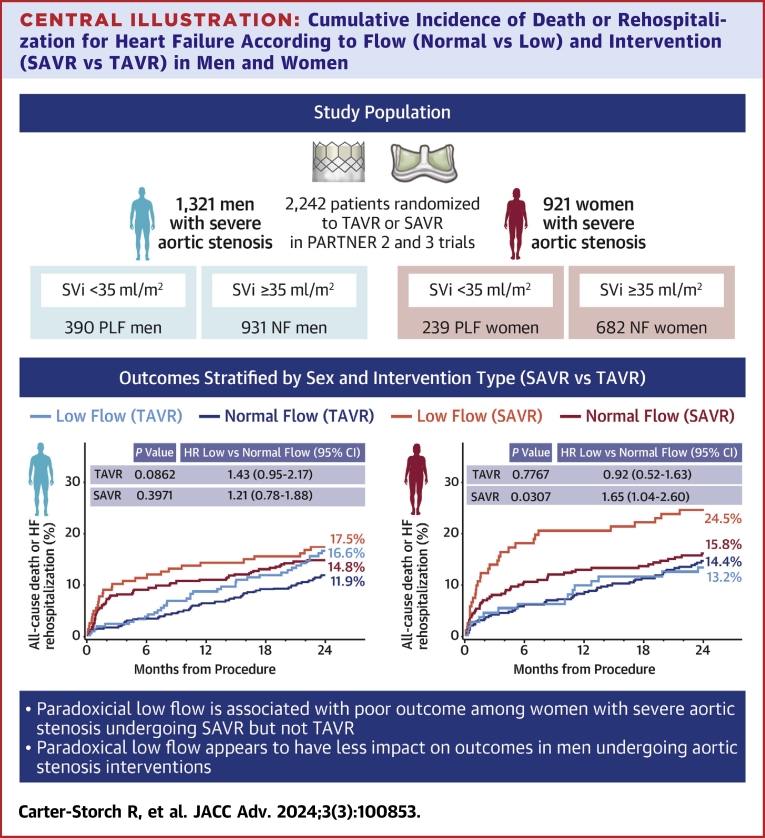
**Cumulative Incidence of Death or Rehospitalization for Heart Failure According to Flow (Normal vs Low) and Intervention (SAVR vs TAVR) in Men and Women** Multivariable logistic regression models: hazard rates are adjusted for age, coronary artery disease, NYHA functional class, hypertension, diabetes, atrial fibrillation, chronic obstructive pulmonary disease, chronic kidney disease, left ventricular ejection fraction, preprocedural aortic valve mean gradient, postprocedural patient-prosthesis mismatch, and transvalvular regurgitation at 30 days. NF = normal flow; PLF = paradoxical low flow; SAVR = surgical aortic valve replacement; TAVR = transcatheter aortic valve replacement.

**Figure 2:**
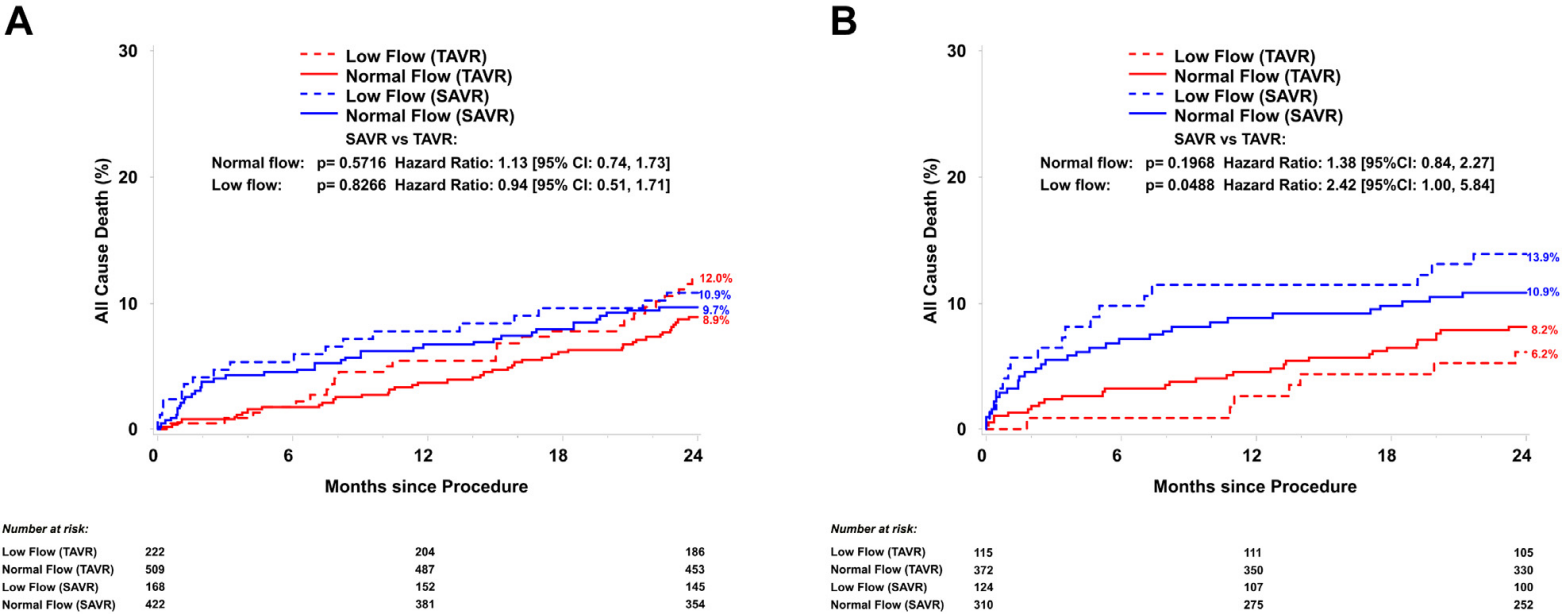
Cumulative Incidence of Mortality Cumulative incidence of death according to flow (normal vs low) and intervention (SAVR vs TAVR) in men (A) and women (B). Multivariate logistic regression models: hazard rates are adjusted for age, coronary artery disease, NYHA functional class, hypertension, diabetes, atrial fibrillation, chronic obstructive pulmonary disease, chronic kidney disease, left ventricular ejection fraction, preprocedural aortic valve mean gradient, postprocedural patient-prosthesis mismatch, and transvalvular regurgitation at 30 days. SAVR = surgical aortic valve replacement; TAVR = transcatheter aortic valve replacement.

Low preprocedural SVi was not a risk factor for 2-year all-cause mortality in the whole cohort, or in any of the 4 groups (according to sex and TAVR/SAVR) (Figure 2). However, the 2-year mortality rate for women with PLF undergoing SAVR was higher compared to women with PLF undergoing TAVR (13.9% vs 6.2%, univariable HR 2.42 [95% CI: 1.00-5.84], *P* = 0.049). When adjusting for known risk factors (age, coronary artery disease, hypertension, diabetes, atrial fibrillation, chronic obstructive pulmonary disease, chronic kidney disease ≥ moderate-severe, LVEF, mean gradient), this difference was no longer significant, but there was a nonsignificant trend toward higher risk of all-cause mortality in women with PLF undergoing SAVR compared to those undergoing TAVR (adjusted HR: 2.33 [95% CI: 0.91-5.98], *P* = 0.08).

## Discussion

This analysis of data from the PARTNER 2 and 3 trials represents one of the largest studies on the impact of sex and preprocedural flow status on outcomes following SAVR and TAVR. Our main findings are that in patients with severe AS and preserved LVEF: 1) PLF was a risk factor for death or heart failure hospitalization among women undergoing SAVR but not TAVR; 2) women with PLF had a trend toward increased risk of all-cause mortality with SAVR compared with TAVR; and 3) PLF was not associated with worse outcomes among men undergoing either SAVR or TAVR.

Reduced SVi is a well-described risk factor for adverse outcomes in AS in observational studies,^12^^,^^21^, ^22^, ^23^, ^24^, ^25^ although this association is attenuated in multivariable adjustment due to differences in comorbidities.^12^^,^^25^ The increased risk of events is, in part, related to adverse LV remodeling with higher relative wall thickness,^9^^,^^10^^,^^24^ more advanced diastolic dysfunction,^24^^,^^26^ and a higher proportion of patients with atrial fibrillation.^13^^,^^22^^,^^27^ Previous studies have mostly focused on PLF or sex as 2 isolated risk factors, whereas in our study, we show that the impact of PLF is not uniform among men and women: that is, TAVR appears to be superior to SAVR in the vulnerable subset of women with PLF, whereas women with NF or men (regardless of flow status) have similar outcomes with SAVR and TAVR. This finding is consistent with a previous study showing that PLF is an independent risk factor for long-term mortality after SAVR, but that this risk factor was only present in women or more prevalent in women.^9^ It highlights the need to stratify AS studies according to sex, because of sex-specific differences in pathophysiology and prognosis.

The PLF group in this study had a higher proportion of diabetes, atrial fibrillation, and worse baseline symptom severity compared to the NF group, all of which are risk factors for worse prognosis after AVR;^10^^,^^13^ these factors may explain, at least in part, the association between PLF and outcomes. However, after multivariable adjustment for known risk factors, PLF remained an independent risk factor among women undergoing SAVR.

The second interesting finding in this article was that among women with PLF, SAVR was associated with a higher 2-year mortality compared to TAVR. This should be interpreted with caution as the number of events in this subgroup was small, and because the association was no longer significant after multivariable adjustment. According to current guidelines from the American College of Cardiology/American Heart Association, both SAVR and transfemoral TAVR can be considered equal treatments and the choice depends on expected longevity of the patient and valve durability.^4^ The guidelines do not take into consideration sex and flow status in the decision process for the management of patients with AS. However, based on our study, PLF could tip the decision in favor of TAVR compared with SAVR for women.

Other studies have found PLF to be a risk factor for worse outcomes after AVR.^9^, ^10^, ^11^^,^^13^^,^^27^ In PLF patients in the PARTNER 1 trial, SAVR resulted in an early increased hazard compared to TAVR, though long-term survival was similar.^11^ This early increase in mortality and in-hospital complications among PLF patients was confirmed in another observational study of patients undergoing SAVR, where known risk scores underestimated the perioperative risk in this group.^10^ Part of the increased surgical risk associated with PLF may be attributable to concomitant risk factors, explaining why the association between PLF and increased long-term mortality after SAVR was no longer significant after multivariable adjustment.^10^

Among AS cohorts, women have worse prognosis than men, in part because they are less likely to be referred to AVR or they are referred later in the course of the disease.^28^, ^29^, ^30^ Furthermore, women have higher 30-day mortality and higher in-hospital complication rates after SAVR than men, even after propensity score matching.^29^^,^^31^ Several factors may contribute to this increased risk in women vs men. In the present study, the prevalence of frailty was higher in women than in men. Frailty has been reported as an independent predictor of outcome after AVR.^32^^,^^33^ The LV remodeling process in women with AS also differs from that in men, with more pronounced LV concentric remodeling and higher extent of diffuse myocardial fibrosis, as was also seen in our sample, where women with PLF had the smallest LV volumes.^34^, ^35^, ^36^

Women also have smaller aortic annuli than men,^12^^,^^37^^,^^38^ increasing their risk for prosthesis-patient mismatch, which may hinder the reverse LV remodeling process, regression of LV diastolic dysfunction, and thus prognosis following AVR.^39^^,^^40^ This issue may be more important with SAVR than TAVR, because of the higher risk of severe prosthesis-patient mismatch associated with SAVR in patients with a small annulus.^40^^,^^41^ In the SAVR arm of the present study, women, especially women with PLF, received smaller valves than men, and they had the lowest AVA, indexed AVA, and the highest proportion of PPM at 1 month. Better effective orifice areas, lower postoperative gradients, and lower incidence of PPM achieved with TAVR may explain, at least in part, the better prognosis associated with TAVR vs SAVR in women.

### Strengths and limitations

By combining data from the PARTNER 2 and 3 studies, we were able to perform one of the largest studies on preprocedural PLF in patients randomized to SAVR or TAVR. Previous studies have mostly been performed on retrospective data with the risk of residual confounding despite multivariable adjustment. Nevertheless, there are some limitations to this study. The first is that enrolled patients had to fulfill the inclusion criteria for the randomized trials, and thus our results may not be generalizable to all patients with AS.

We excluded patients with LVEF <50% and our conclusions can therefore not be extrapolated to patients with classical low flow. Furthermore, in the PARTNER 2 and 3 trials, patients with mean gradient <40 mm Hg measured at rest at the recruitment sites were excluded, and our cohort therefore mostly consists of patients with low flow, high gradient AS. Although a number of patients with mean gradient <40 mm Hg measured by the core lab are present, the sickest PLF patients (ie, the patients with the lowest gradients) were not included in the PARTNER trials. However, as a significant proportion of patients with PLF, low gradient according to core lab measurements are present in this study and given that the difference in outcomes should be more pronounced in sicker patients, we believe our results are applicable to all women with low flow and either high or low gradient. In men, the absence of difference could be debatable and different in patients with more pronounced low gradient. Also, we did not test the possibility of sex-specific SVi thresholds^9^ to define low flow, due to the number of studied groups.

We were only able to include 2-year outcomes, so long-term outcomes are still unknown, and should be studied. Furthermore, patient-centered outcomes and functional outcomes were not analyzed, and could be a topic for further studies.

Finally, as mentioned previously, the number of deaths was low in the sample, especially when looking at smaller subgroups, and our results regarding higher mortality for SAVR vs TAVR for women with PLF should therefore be considered hypothesis generating.

## Conclusions

In the PARTNER 2 and 3 trials, PLF pattern was associated with worse outcomes in women undergoing SAVR but not TAVR. Moreover, among women with PLF, SAVR may possibly be associated with higher rates of 2-year mortality compared to TAVR. In men undergoing either SAVR or TAVR, PLF was not associated with worse outcomes.

These findings suggest that even among patients with preserved LV function, sex and flow status should be taken into account in the decision-making process between TAVR vs SAVR and that TAVR may potentially be preferred over SAVR in women with PLF. Further randomized studies are needed to confirm the superiority of TAVR over SAVR in this particular subset of patients.

PERSPECTIVES **COMPETENCY IN PATIENT CARE AND PROCEDURAL SKILLS:** PLF among women with severe AS is associated with worse prognosis for SAVR but not TAVR. Furthermore, among women with PLF, SAVR is associated with a higher 2-year mortality compared to TAVR.

**TRANSLATIONAL OUTLOOK:** This study suggests that TAVR should be preferred over SAVR in women with PLF. Further randomized studies are needed, however, to confirm this finding.

## Funding support and author disclosures

Statistical analyses were performed by employees of Edwards Lifesciences. Dr Carter-Storch has received a travel grant from AstraZeneca. Dr Hahn has received speaker fees from Abbott Structural, Baylis Medical, Edwards Lifesciences, and Philips Healthcare; has institutional consulting contracts for which she receives no direct compensation with Abbott Structural, Edwards Lifesciences, Medtronic, and Novartis; and is Chief Scientific Officer for the Echocardiography Core Laboratory at the Cardiovascular Research Foundation for multiple industry-sponsored tricuspid valve trials, for which she receives no direct industry compensation. Dr Abbas has received research support from and consulting for Edwards Lifesciences. Dr Mack has served as co-primary investigator for the PARTNER Trial for Edwards Lifesciences and the COAPT trial for Abbott; and has served as study chair for the APOLLO trial for Medtronic; no direct compensation for any of these activities. Dr Leon has received institutional research support from Edwards Lifesciences, Medtronic, Boston Scientific, and Abbott; and consulting/advisory board participation for Medtronic, Boston Scientific, Gore, Meril Lifescience, and Abbott. Dr Pibarot has received funding from Edwards Lifesciences, Medtronic, Pi-Cardia, and Cardiac Phoenix for echocardiography core laboratory analyses and research studies in the field of transcatheter valve therapies, for which he received no personal compensation; and has received lecture fees from Edwards Lifesciences and Medtronic. Dr Clavel has received core laboratory contract with Edwards Lifesciences; and research grants with Edwards Lifesciences and Medtronic, both without direct compensation. The PARTNER trials were funded by Edwards Lifesciences. All other authors have reported that they have no relationships relevant to the contents of this paper to disclose.
