# Supplementary material for: Effect of Sex and Flow Status on Outcomes After Surgical or Transcatheter Aortic Valve Replacement
Source: JACC Adv. 2024 Feb 16;3(3):100853. doi: 10.1016/j.jacadv.2024.100853 (PMC11198680; doi:10.1016/j.jacadv.2024.100853)
Supplement: Supplemental Tables 1-8 [file mmc1.docx]

| Supplemental Table 1.1: Baseline Characteristics by Sex in Total Sample | | | | |
| --- | --- | --- | --- | --- |
|  | **Total (N= 2242)** | **Men (N= 1321)** | **Women (N= 921)** | **p value** |
| Age (years) | 78.9±7.45 (2242) | 78.3±7.63 (1321) | 79.8±7.11 (921) | <0.0001 |
| BMI (kg/m^2^) | 29.4±5.92 (2242) | 29.3±5.10 (1321) | 29.5±6.93 (921) | 0.39 |
| BSA (m^2^) | 1.9±0.24 (2242) | 2.1±0.19 (1321) | 1.8±0.20 (921) | <0.0001 |
| STS Score (%) | 4.2 [2.2, 5.6] | 4.0 [1.8, 5.2] | 4.7 [2.8, 6.1] | <0.0001 |
| NYHA Class | | | | |
| I | 0.4% (8/2241) | 0.5% (6/1320) | 0.2% (2/921) | 0.0005 |
| II | 45.0% (1008/2241) | 48.5% (640/1320) | 40.0% (368/921) |  |
| III | 45.7% (1024/2241) | 42.3% (559/1320) | 50.5% (465/921) |  |
| IV | 9.0% (201/2241) | 8.7% (115/1320) | 9.3% (86/921) |  |
| KCCQ Overall Summary Score | 62.5 [44.8, 80.0] | 65.7 [48.4, 82.3] | 57.8 [40.6, 75.3] | <0.0001 |
| Diabetes Mellitus | 31.9% (715/2241) | 34.2% (452/1321) | 28.6% (263/920) | 0.005 |
| Previous or Current Smoker | 49.8% (1117/2242) | 60.6% (801/1321) | 34.3% (316/921) | <0.0001 |
| CAD | 51.2% (1147/2240) | 58.2% (768/1319) | 41.2% (379/921) | <0.0001 |
| Prior MI | 10.4% (233/2240) | 12.5% (165/1319) | 7.4% (68/921) | <0.0001 |
| Prior PCI | 23.9% (536/2238) | 28.2% (372/1319) | 17.8% (164/919) | <0.0001 |
| Prior CABG | 16.1% (361/2237) | 22.9% (302/1319) | 6.4% (59/918) | <0.0001 |
| PVD | 21.2% (475/2239) | 23.5% (310/1319) | 17.9% (165/920) | 0.002 |
| Prior Stroke | 7.3% (164/2242) | 7.7% (102/1321) | 6.7% (62/921) | 0.3760 |
| Atrial fibrillation | 27.9% (626/2241) | 31.4% (415/1320) | 22.9% (211/921) | <0.0001 |
| Permanent Pacemaker | 7.4% (166/2242) | 7.8% (103/1321) | 6.8% (63/921) | 0.39 |
| Hypertension | 90.4% (2024/2240) | 89.5% (1181/1320) | 91.6% (843/920) | 0.09 |
| Dyslipidemia | 66.4% (1488/2242) | 70.4% (930/1321) | 60.6% (558/921) | <0.0001 |
| COPD | 20.2% (452/2234) | 21.2% (279/1317) | 18.9% (173/917) | 0.18 |
| CKD | | | | |
| Normal or high | 13.8% (306/2212) | 14.8% (193/1305) | 12.5% (113/907) | <0.0001 |
| Mildly decreased | 45.9% (1016/2212) | 49.6% (647/1305) | 40.7% (369/907) |  |
| Mildly to moderately decreased | 25.5% (565/2212) | 25.0% (326/1305) | 26.4% (239/907) |  |
| Moderately to severely decreased | 12.4% (275/2212) | 9.0% (118/1305) | 17.3% (157/907) |  |
| Severely decreased | 2.3% (50/2212) | 1.6% (21/1305) | 3.2% (29/907) |  |
| Kidney failure | 0.0% (0/2212) | 0.0% (0/1305) | 0.0% (0/907) |  |
| Creatinine > 2mg/dL | 3.7% (83/2242) | 4.0% (53/1321) | 3.3% (30/921) | 0.35 |
| Liver disease | 0.6% (14/2241) | 0.8% (11/1321) | 0.3% (3/920) | 0.13 |
| Frailty | 3.6% (80/2240) | 2.3% (30/1320) | 5.4% (50/920) | <0.0001 |
| Note: Summary statistics:  Categorical measures-% (No. / Total no.)  Continuous measures – mean ± SD (n); median (Q1, Q3); [min, max]  Two sample t tests were used for continous variables. Chisq tests were used for categorical variables. | | | | |

| Supplemental Table 1.2 Baseline echo Information by Sex in Total Sample | | | | |
| --- | --- | --- | --- | --- |
|  | **Total (N= 2242)** | **Men (N= 1321)** | **Women (N= 921)** | **p value** |
| Stroke volume (mL) | 77.6±17.25 (2242) | 81.7±17.22 (1321) | 71.8±15.54 (921) | <0.0001 |
| Stroke volume index (mL/m^2^) | 40.3±8.70 (2242) | 40.0±8.73 (1321) | 40.8±8.65 (921) | 0.04 |
| AoV mean gradient (mmHg) | 47.4±12.26 (2239) | 47.1±11.75 (1320) | 47.9±12.95 (919) | 0.10 |
| AoV peak gradient (mmHg) | 79.3±19.51 (2239) | 78.5±18.56 (1320) | 80.4±20.75 (919) | 0.03 |
| AV peak velocity (m/s) | 442.0±52.81 (2239) | 440.1±50.53 (1320) | 444.7±55.84 (919) | 0.04 |
| LVEF (Simpson/visually estimated, %) | 64.0±7.29 (2242) | 63.4±7.00 (1321) | 64.9±7.60 (921) | <0.0001 |
| LV mass (g) | 381.1±211.12 (2084) | 409.3±228.63 (1237) | 339.8±174.67 (847) | <0.0001 |
| LV mass index (g/m^2^) | 198.8±108.24 (2084) | 202.2±114.87 (1237) | 193.8±97.62 (847) | 0.08 |
| LVEDD (mm) | 4.7±0.58 (2140) | 4.9±0.53 (1268) | 4.4±0.54 (872) | <0.0001 |
| LVESD (mm) | 3.0±0.59 (2104) | 3.1±0.58 (1251) | 2.7±0.54 (853) | <0.0001 |
| LVEDV (mL) | 99.8±28.90 (1956) | 110.6±28.32 (1170) | 83.7±21.23 (786) | <0.0001 |
| LVESV (mL) | 35.7±14.21 (1953) | 40.2±14.45 (1169) | 28.8±10.65 (784) | <0.0001 |
| AV area (cm^2^) | 0.7±0.17 (2239) | 0.8±0.17 (1320) | 0.7±0.15 (919) | <0.0001 |
| AV area index (cm^2^/m^2^) | 0.4±0.09 (2239) | 0.4±0.09 (1320) | 0.4±0.09 (919) | 0.99 |
| DVI | 0.2±0.05 (2239) | 0.2±0.04 (1320) | 0.2±0.05 (919) | <0.0001 |
| Moderate+ aortic regurgitation | 6.7% (149/2221) | 6.4% (84/1308) | 7.1% (65/913) | 0.52 |
| Moderate+ mitral regurgitation | 7.0% (154/2186) | 5.5% (71/1286) | 9.2% (83/900) | 0.0009 |
| Moderate+ tricuspid regurgitation | 7.3% (155/2130) | 5.9% (74/1253) | 9.2% (81/877) | 0.004 |
| Note: Summary statistics:  Categorical measures-% (No. / Total no.)  Continuous measures – mean ± SD (n); median (Q1, Q3); [min, max] | | | | |

| Supplemental Table 2.1 Baseline Characteristics by SVi | | | | |
| --- | --- | --- | --- | --- |
|  | **Total (N= 2242)** | **SVI < 35 mL/m^2^ (N= 629)** | **SVI ≥ 35 mL/m^2^ (N= 1613)** | **p value** |
| Age (years) | 78.9±7.45 (2242) | 78.8±7.66 (629) | 79.0±7.37 (1613) | 0.71 |
| Sex(male) | 58.9% (1321/2242) | 62.0% (390/629) | 57.7% (931/1613) | 0.06 |
| BMI (kg/m^2^) | 29.4±5.92 (2242) | 30.7±6.29 (629) | 28.8±5.69 (1613) | <0.0001 |
| BSA (m^2^) | 1.9±0.24 (2242) | 2.0±0.25 (629) | 1.9±0.24 (1613) | <0.0001 |
| STS Score (%) | 4.2 [2.2, 5.6] | 4.4 [2.4, 5.8] | 4.2 [2.1, 5.5] | 0.001 |
| NYHA Class | | | | |
| I | 0.4% (8/2241) | 0.2% (1/629) | 0.4% (7/1612) | 0.001 |
| II | 45.0% (1008/2241) | 39.4% (248/629) | 47.1% (760/1612) |  |
| III | 45.7% (1024/2241) | 48.6% (306/629) | 44.5% (718/1612) |  |
| IV | 9.0% (201/2241) | 11.8% (74/629) | 7.9% (127/1612) |  |
| KCCQ Overall Summary Score | 62.5 [44.8, 80.0] | 58.5 [39.8, 75.0] | 64.1 [46.6, 81.8] | <0.0001 |
| Diabetes Mellitus | 31.9% (715/2241) | 38.8% (244/629) | 29.2% (471/1612) | <0.0001 |
| Previous or Current Smoker | 49.8% (1117/2242) | 50.2% (316/629) | 49.7% (801/1613) | 0.81 |
| CAD | 51.2% (1147/2240) | 55.0% (346/629) | 49.7% (801/1611) | 0.02 |
| Prior MI | 10.4% (233/2240) | 8.9% (56/629) | 11.0% (177/1611) | 0.15 |
| Prior PCI | 23.9% (536/2238) | 24.0% (151/628) | 23.9% (385/1610) | 0.95 |
| Prior CABG | 16.1% (361/2237) | 18.5% (116/628) | 15.2% (245/1609) | 0.06 |
| PVD | 21.2% (475/2239) | 23.4% (147/627) | 20.3% (328/1612) | 0.1074 |
| Prior Stroke | 7.3% (164/2242) | 7.8% (49/629) | 7.1% (115/1613) | 0.59 |
| Atrial fibrillation | 27.9% (626/2241) | 38.6% (243/629) | 23.8% (383/1612) | <0.0001 |
| Permanent Pacemaker | 7.4% (166/2242) | 11.9% (75/629) | 5.6% (91/1613) | <0.0001 |
| Hypertension | 90.4% (2024/2240) | 89.7% (564/629) | 90.6% (1460/1611) | 0.49 |
| Dyslipidemia | 66.4% (1488/2242) | 65.0% (409/629) | 66.9% (1079/1613) | 0.40 |
| COPD | 20.2% (452/2234) | 22.7% (143/629) | 19.3% (309/1605) | 0.07 |
| CKD | | | | |
| Normal or high | 13.8% (306/2212) | 12.4% (77/620) | 14.4% (229/1592) | 0.14 |
| Mildly decreased | 45.9% (1016/2212) | 45.8% (284/620) | 46.0% (732/1592) |  |
| Mildly to moderately decreased | 25.5% (565/2212) | 28.9% (179/620) | 24.2% (386/1592) |  |
| Moderately to severely decreased | 12.4% (275/2212) | 10.8% (67/620) | 13.1% (208/1592) |  |
| Severely decreased | 2.3% (50/2212) | 2.1% (13/620) | 2.3% (37/1592) |  |
| Kidney failure | 0.0% (0/2212) | 0.0% (0/620) | 0.0% (0/1592) |  |
| Creatinine > 2mg/dL | 3.7% (83/2242) | 2.9% (18/629) | 4.0% (65/1613) | 0.19 |
| Liver disease | 0.6% (14/2241) | 0.8% (5/628) | 0.6% (9/1613) | 0.52 |
| Frailty | 3.6% (80/2240) | 3.3% (21/628) | 3.7% (59/1612) | 0.72 |
| Note: Summary statistics:  Categorical measures-% (No. / Total no.)  Continuous measures – mean ± SD (n); median (Q1, Q3); [min, max]  Two sample t tests were used for continous variables. Chisq tests were used for categorical variables. | | | | |

| Suppl Table 2.2 Baseline Echo Information by Guideline Recommended Low Flow | | | | |
| --- | --- | --- | --- | --- |
|  | **Total (N= 2242)** | **SVI < 35 mL/m^2^ (N= 629)** | **SVI ≥ 35 mL/m^2^ (N= 1613)** | **p value** |
| Stroke volume (mL) | 77.6±17.25 (2242) | 61.5±9.95 (629) | 83.9±15.29 (1613) | <0.0001 |
| Stroke volume index (mL/m^2^) | 40.3±8.70 (2242) | 30.7±3.36 (629) | 44.1±7.14 (1613) | <0.0001 |
| AoV mean gradient (mmHg) | 47.4±12.26 (2239) | 44.8±11.61 (628) | 48.4±12.36 (1611) | <0.0001 |
| AoV peak gradient (mmHg) | 79.3±19.51 (2239) | 74.8±18.36 (628) | 81.0±19.67 (1611) | <0.0001 |
| AV peak velocity (m/s) | 442.0±52.81 (2239) | 429.4±50.97 (628) | 446.9±52.71 (1611) | <0.0001 |
| LVEF (Simpson/visually estimated, %) | 64.0±7.29 (2242) | 62.1±7.28 (629) | 64.7±7.16 (1613) | <0.0001 |
| LV mass (g) | 381.1±211.12 (2084) | 393.5±205.95 (576) | 376.3±212.94 (1508) | 0.09 |
| LV mass index (g/m^2^) | 198.8±108.24 (2084) | 199.6±103.27 (576) | 198.5±110.12 (1508) | 0.82 |
| LVEDD (mm) | 4.7±0.58 (2140) | 4.6±0.53 (594) | 4.8±0.59 (1546) | <0.0001 |
| LVESD (mm) | 3.0±0.59 (2104) | 3.0±0.59 (587) | 3.0±0.60 (1517) | 0.81 |
| LVEDV (mL) | 99.8±28.90 (1956) | 93.9±27.03 (526) | 101.9±29.28 (1430) | <0.0001 |
| LVESV (mL) | 35.7±14.21 (1953) | 35.4±13.67 (525) | 35.8±14.40 (1428) | 0.61 |
| AV area (cm^2^) | 0.7±0.17 (2239) | 0.6±0.14 (628) | 0.8±0.17 (1611) | <0.0001 |
| AV area index (cm^2^/m^2^) | 0.4±0.09 (2239) | 0.3±0.06 (628) | 0.4±0.08 (1611) | <0.0001 |
| DVI | 0.2±0.05 (2239) | 0.2±0.04 (628) | 0.2±0.04 (1611) | <0.0001 |
| Moderate+ aortic regurgitation | 6.7% (149/2221) | 2.9% (18/619) | 8.2% (131/1602) | <0.0001 |
| Moderate+ mitral regurgitation | 7.0% (154/2186) | 8.3% (50/606) | 6.6% (104/1580) | 0.17 |
| Moderate+ tricuspid regurgitation | 7.3% (155/2130) | 9.8% (57/583) | 6.3% (98/1547) | 0.006 |
| Note: Summary statistics:  Categorical measures-% (No. / Total no.)  Continuous measures – mean ± SD (n); median (Q1, Q3); [min, max] | | | | |

| Supplemental Table 3: Baseline Characteristics by Sex and SVI in Total Sample | | | | | | |
| --- | --- | --- | --- | --- | --- | --- |
|  | | **Men (N= 1321)** | | **Women (N= 921)** | |  |
|  | **Total (N= 2242)** | **SVI<35 (N=390)** | **SVI>=35 (N= 931)** | **SVI<35 (N= 239)** | **SVI>=35 (N= 682)** | **p value** |
| Age (years) | 78.9±7.45 (2242) | 78.4±7.92 (390) | 78.3±7.51 (931) | 79.6±7.19 (239) | 79.9±7.08 (682) | <0.0001 |
| BMI (kg/m^2^) | 29.4±5.92 (2242) | 30.5±5.53 (390) | 28.8±4.82 (931) | 31.1±7.35 (239) | 29.0±6.69 (682) | <0.0001 |
| BSA (m^2^) | 1.9±0.24 (2242) | 2.1±0.20 (390) | 2.0±0.19 (931) | 1.8±0.21 (239) | 1.8±0.20 (682) | <0.0001 |
| STS Score (%) | 4.2 [2.2, 5.6] | 4.2 [2.2, 5.6] | 3.4 [1.7, 5.1] | 4.7 [3.4, 6.3] | 4.7 [2.7, 6.1] | <0.0001 |
| I | 0.4% (8/2241) | 0.3% (1/390) | 0.5% (5/930) | 0.0% (0/239) | 0.3% (2/682) | <0.0001 |
| II | 45.0% (1008/2241) | 43.3% (169/390) | 50.6% (471/930) | 33.1% (79/239) | 42.4% (289/682) | <0.0001 |
| III | 45.7% (1024/2241) | 45.4% (177/390) | 41.1% (382/930) | 54.0% (129/239) | 49.3% (336/682) | <0.0001 |
| IV | 9.0% (201/2241) | 11.0% (43/390) | 7.7% (72/930) | 13.0% (31/239) | 8.1% (55/682) | <0.0001 |
| KCCQ Overall Summary Score | 62.5 [44.8, 80.0] | 61.1 [43.2, 76.8] | 68.0 [51.0, 84.7] | 53.4 [37.8, 70.4] | 58.7 [42.7, 77.0] | <0.0001 |
| Diabetes Mellitus | 31.9% (715/2241) | 41.5% (162/390) | 31.1% (290/931) | 34.3% (82/239) | 26.6% (181/681) | <0.0001 |
| Previous or Current Smoker | 49.8% (1117/2242) | 60.8% (237/390) | 60.6% (564/931) | 33.1% (79/239) | 34.8% (237/682) | <0.0001 |
| CAD | 51.2% (1147/2240) | 61.3% (239/390) | 56.9% (529/929) | 44.8% (107/239) | 39.9% (272/682) | <0.0001 |
| Prior MI | 10.4% (233/2240) | 9.7% (38/390) | 13.7% (127/929) | 7.5% (18/239) | 7.3% (50/682) | 0.0002 |
| Prior PCI | 23.9% (536/2238) | 27.2% (106/390) | 28.6% (266/929) | 18.9% (45/238) | 17.5% (119/681) | <0.0001 |
| Prior CABG | 16.1% (361/2237) | 25.6% (100/390) | 21.7% (202/929) | 6.7% (16/238) | 6.3% (43/680) | <0.0001 |
| PVD | 21.2% (475/2239) | 24.7% (96/389) | 23.0% (214/930) | 21.4% (51/238) | 16.7% (114/682) | 0.005 |
| Prior Stroke | 7.3% (164/2242) | 9.5% (37/390) | 7.0% (65/931) | 5.0% (12/239) | 7.3% (50/682) | 0.19 |
| Atrial fibrillation | 27.9% (626/2241) | 43.3% (169/390) | 26.5% (246/930) | 31.0% (74/239) | 20.1% (137/682) | <0.0001 |
| Permanent Pacemaker | 7.4% (166/2242) | 12.6% (49/390) | 5.8% (54/931) | 10.9% (26/239) | 5.4% (37/682) | <0.0001 |
| Hypertension | 90.4% (2024/2240) | 89.5% (349/390) | 89.5% (832/930) | 90.0% (215/239) | 92.2% (628/681) | 0.27 |
| Dyslipidemia | 66.4% (1488/2242) | 69.2% (270/390) | 70.9% (660/931) | 58.2% (139/239) | 61.4% (419/682) | <0.0001 |
| COPD | 20.2% (452/2234) | 25.4% (99/390) | 19.4% (180/927) | 18.4% (44/239) | 19.0% (129/678) | 0.048 |
| Normal or high | 13.8% (306/2212) | 11.7% (45/383) | 16.1% (148/922) | 13.5% (32/237) | 12.1% (81/670) | <0.0001 |
| Mildly decreased | 45.9% (1016/2212) | 48.8% (187/383) | 49.9% (460/922) | 40.9% (97/237) | 40.6% (272/670) | <0.0001 |
| Mildly to moderately decreased | 25.5% (565/2212) | 30.5% (117/383) | 22.7% (209/922) | 26.2% (62/237) | 26.4% (177/670) | <0.0001 |
| Moderately to severely decreased | 12.4% (275/2212) | 7.6% (29/383) | 9.7% (89/922) | 16.0% (38/237) | 17.8% (119/670) | <0.0001 |
| Severely decreased | 2.3% (50/2212) | 1.3% (5/383) | 1.7% (16/922) | 3.4% (8/237) | 3.1% (21/670) | <0.0001 |
| Kidney failure | 0.0% (0/2212) | 0.0% (0/383) | 0.0% (0/922) | 0.0% (0/237) | 0.0% (0/670) | <0.0001 |
| Creatinine > 2mg/dL | 3.7% (83/2242) | 2.8% (11/390) | 4.5% (42/931) | 2.9% (7/239) | 3.4% (23/682) | 0.37 |
| Liver disease | 0.6% (14/2241) | 1.3% (5/390) | 0.6% (6/931) | 0.0% (0/238) | 0.4% (3/682) | 0.20 |
| Frailty | 3.6% (80/2240) | 2.1% (8/389) | 2.4% (22/931) | 5.4% (13/239) | 5.4% (37/681) | 0.001 |
| Note: Summary statistics:           Categorical measures-% (No. / Total no.)           Continuous measures – mean ± SD (n); median (Q1, Q3); [min, max] | | | | | | |

| Supplemental Table 4: Multivariable Cox Model for Death/HF Hospitalization - All Patients | | |
| --- | --- | --- |
| **Parameter** | **HRs (95% CI)** | **p-value*** |
| SVi, <35ml/m^2^ vs. >=35ml/m^2^ | 1.094 (0.821, 1.457) | 0.54 |
| Sex, male vs. female | 0.877 (0.664, 1.159) | 0.36 |
| Treatment, TAVR vs. SAVR | 1.089 (0.832, 1.424) | 0.54 |
| Age, per 1 year increase | 1.043 (1.022, 1.065) | <0.0001 |
| CAD, Yes vs. No | 1.048 (0.798, 1.378) | 0.73 |
| NYHA Class III/IV, Yes vs. No | 1.213 (0.911, 1.614) | 0.19 |
| HTN, Yes vs. No | 1.014 (0.634, 1.623) | 0.95 |
| Diabetes, Yes vs. No | 1.001 (0.747, 1.342) | 0.99 |
| Afib, Yes vs. No | 2.063 (1.576, 2.702) | <.0001 |
| COPD, Yes vs. No | 1.472 (1.099, 1.971) | 0.01 |
| CKD, moderately to severely decreased or severely decreased vs. other CKD | 1.088 (0.765, 1.547) | 0.64 |
| LVEF, per 1% increase | 0.976 (0.957, 0.995) | 0.02 |
| AoV mean gradient, per 1 mmHg increase | 0.986 (0.975, 0.998) | 0.02 |
| Patient-prothesis mismatch, Yes vs. No | 0.856 (0.621, 1.180) | 0.34 |
| Transvalvular regurgitation at 30 days, Mild+ vs. None/Trace | 1.457 (0.597, 3.560) | 0.41 |
| 30-day landmark analysis adjusted for PPM and 30-day transvalvular regurgitation | | |

| Supplemental Table 5: Multivariable Cox Model for Death/HF Hospitalization - Male TAVR patients | | |
| --- | --- | --- |
| **Parameter** | **HRs (95% CI)** | **p-value*** |
| SVi, <35ml/m^2^ vs. >=35ml/m^2^ | 1.092 (0.679, 1.756) | 0.72 |
| Age, per 1 year increase | 1.035 (1.000, 1.071) | 0.05 |
| CAD, Yes vs. No | 1.448 (0.882, 2.374) | 0.14 |
| NYHA Class III/IV, Yes vs. No | 1.850 (1.105, 3.097) | 0.02 |
| HTN, Yes vs. No | 0.632 (0.325, 1.228) | 0.18 |
| Diabetes, Yes vs. No | 0.765 (0.457, 1.281) | 0.31 |
| Afib, Yes vs. No | 2.240 (1.405, 3.570) | 0.0007 |
| COPD, Yes vs. No | 2.008 (1.269, 3.177) | 0.003 |
| CKD, moderately to severely decreased or severely decreased vs. other CKD | 1.325 (0.691, 2.541) | 0.40 |
| LVEF, per 1% increase | 0.987 (0.955, 1.021) | 0.46 |
| AoV mean gradient, per 1 mmHg increase | 0.986 (0.965, 1.007) | 0.19 |
| Patient-prothesis mismatch, Yes vs. No | 0.606 (0.315, 1.165) | 0.13 |
| Transvalvular regurgitation at 30 days, Mild+ vs. None/Trace | 1.798 (0.519, 6.225) | 0.35 |
| 30-day landmark analysis adjusted for PPM and 30-day transvalvular regurgitation | | |

| Supplemental Table 6: Multivariable Cox Model for Death/HF Hospitalization - Male SAVR patients | | |
| --- | --- | --- |
| **Parameter** | **HRs (95% CI)** | **p-value*** |
| SVi, <35ml/m^2^ vs. >=35ml/m^2^ | 0.762 (0.406, 1.430) | 0.40 |
| Age, per 1 year increase | 1.073 (1.025, 1.123) | 0.003 |
| CAD, Yes vs. No | 1.195 (0.637, 2.244) | 0.58 |
| NYHA Class III/IV, Yes vs. No | 0.941 (0.525, 1.686) | 0.84 |
| HTN, Yes vs. No | 1.477 (0.506, 4.315) | 0.48 |
| Diabetes, Yes vs. No | 0.807 (0.440, 1.480) | 0.49 |
| Afib, Yes vs. No | 2.617 (1.493, 4.587) | 0.0008 |
| COPD, Yes vs. No | 1.020 (0.542, 1.920) | 0.95 |
| CKD, moderately to severely decreased or severely decreased vs. other CKD | 0.896 (0.345, 2.324) | 0.82 |
| LVEF, per 1% increase | 0.950 (0.907, 0.994) | 0.03 |
| AoV mean gradient, per 1 mmHg increase | 0.965 (0.937, 0.994) | 0.02 |
| Patient-prothesis mismatch, Yes vs. No | 1.063 (0.540, 2.094) | 0.86 |
| Transvalvular regurgitation at 30 days, Mild+ vs. None/Trace | 0.618 (0.084, 4.576) | 0.64 |
| 30-day landmark analysis adjusted for PPM and 30-day transvalvular regurgitation Source: Table 7.2.1.5 based on data extracted on P3 (08OCT2020), P2 SAVR (21JUN2021) and S3I (23APR2021) and run on 04OCT2022 (T09:14) using ADECHO, ADTTE, ADSL. Path: N:\9999-Pooled Analysis\2021\Low flow\pgm\tlf\t_7xxx.sas | | |

| Supplemental Table 7: Multivariable Cox Model for Death/HF Hospitalization - Female TAVR patients | | |
| --- | --- | --- |
| **Parameter** | **HRs (95% CI)** | **p-value*** |
| SVi, <35ml/m^2^ vs. >=35ml/m^2^ | 0.854 (0.436, 1.670) | 0.64 |
| Age, per 1 year increase | 1.035 (0.990, 1.081) | 0.13 |
| CAD, Yes vs. No | 0.578 (0.320, 1.041) | 0.07 |
| NYHA Class III/IV, Yes vs. No | 1.124 (0.634, 1.996) | 0.69 |
| HTN, Yes vs. No | 1.219 (0.421, 3.526) | 0.72 |
| Diabetes, Yes vs. No | 1.539 (0.846, 2.798) | 0.16 |
| Afib, Yes vs. No | 1.834 (1.004, 3.351) | 0.048 |
| COPD, Yes vs. No | 0.918 (0.445, 1.893) | 0.82 |
| CKD, moderately to severely decreased or severely decreased vs. other CKD | 1.419 (0.761, 2.646) | 0.27 |
| LVEF, per 1% increase | 0.980 (0.943, 1.017) | 0.28 |
| AoV mean gradient, per 1 mmHg increase | 0.986 (0.962, 1.010) | 0.24 |
| Patient-prothesis mismatch, Yes vs. No | 1.014 (0.543, 1.894) | 0.97 |
| Transvalvular regurgitation at 30 days, Mild+ vs. None/Trace | 0.000 (0.000, .) | 0.99 |
| 30-day landmark analysis adjusted for PPM and 30-day transvalvular regurgitation Source: Table 7.2.1.6 based on data extracted on P3 (08OCT2020), P2 SAVR (21JUN2021) and S3I (23APR2021) and run on 04OCT2022 (T09:14) using ADECHO, ADTTE, ADSL. Path: N:\9999-Pooled Analysis\2021\Low flow\pgm\tlf\t_7xxx.sas | | |

| Supplemental Table 8: Multivariable Cox Model for Death/HF Hospitalization - Female SAVR patients | | |
| --- | --- | --- |
| **Parameter** | **HRs (95% CI)** | **p-value*** |
| SVi, <35ml/m^2^ vs. >=35ml/m^2^ | 2.248 (1.142, 4.425) | 0.02 |
| Age, per 1 year increase | 1.018 (0.963, 1.075) | 0.53 |
| CAD, Yes vs. No | 1.048 (0.557, 1.973) | 0.88 |
| NYHA Class III/IV, Yes vs. No | 0.798 (0.391, 1.631) | 0.54 |
| HTN, Yes vs. No | 3.311 (0.447, 24.512) | 0.24 |
| Diabetes, Yes vs. No | 1.110 (0.555, 2.217) | 0.77 |
| Afib, Yes vs. No | 1.505 (0.764, 2.964) | 0.24 |
| COPD, Yes vs. No | 1.729 (0.797, 3.749) | 0.17 |
| CKD, moderately to severely decreased or severely decreased vs. other CKD | 0.726 (0.328, 1.606) | 0.43 |
| LVEF, per 1% increase | 0.961 (0.912, 1.013) | 0.14 |
| AoV mean gradient, per 1 mmHg increase | 1.010 (0.987, 1.034) | 0.40 |
| Patient-prothesis mismatch, Yes vs. No | 0.803 (0.401, 1.608) | 0.54 |
| Transvalvular regurgitation at 30 days, Mild+ vs. None/Trace | 2.864 (0.370, 22.166) | 0.31 |
| 30-day landmark analysis adjusted for PPM and 30-day transvalvular regurgitation Source: Table 7.2.1.7 based on data extracted on P3 (08OCT2020), P2 SAVR (21JUN2021) and S3I (23APR2021) and run on 04OCT2022 (T09:14) using ADECHO, ADTTE, ADSL. Path: N:\9999-Pooled Analysis\2021\Low flow\pgm\tlf\t_7xxx.sas | | |
